# Altered Urine Microbiome in Male Children and Adolescents with Attention-Deficit Hyperactivity Disorder

**DOI:** 10.3390/microorganisms11082063

**Published:** 2023-08-11

**Authors:** Yoon Jae Cho, Bokyoung Shin, Sung-Ha Lee, Sangmin Park, Yoon-Keun Kim, Jae-Jin Kim, Eunjoo Kim

**Affiliations:** 1 Department of Psychiatry, College of Medicine, Yonsei University, Seoul 06273, Republic of Korea; 2 Center for Happiness Studies, Seoul National University, Seoul 08826, Republic of Korea; 3 Interdisciplinary Program in Neuroscience, Seoul National University, Seoul 08826, Republic of Korea; 4 MD Healthcare Inc., Seoul 03923, Republic of Korea; 5 Institute of Behavioral Science in Medicine, Yonsei University College of Medicine, Seoul 06273, Republic of Korea

**Keywords:** attention-deficit hyperactivity disorder, ADHD, microbiome, urobiome

## Abstract

While interest in developing the human microbiome as a biomarker for attention-deficit hyperactivity disorder (ADHD) is increasing, there has been limited exploration in utilizing urine samples. In this study, we analysed urine microbiome profiles by extracting 16S ribosomal DNA from purified bacteria-derived extracellular membrane vesicles obtained from urine samples. Sequencing libraries were constructed by amplifying V3–V4 hypervariable regions sequenced using Illumina MiSeq. Profiles of male Korean children and adolescents with ADHD (*n* = 33) were compared with healthy sex-matched controls (*n* = 39). Statistically controlling for age, we found decreased alpha diversity in the urine bacteria of the ADHD group, as evidenced by reduced Shannon and Simpson indices (*p* < 0.05), and significant differences in beta diversity between the two groups (*p* < 0.001). The phyla *Firmicutes* and *Actinobacteriota*, as well as the genera *Ralstonia* and *Afipia*, were relatively more abundant in the ADHD group. The phylum *Proteobacteria* and the genera *Corynebacterium* and *Peptoniphilus* were more abundant in the control group. Notably, the genus *Afipia* exhibited significant correlations with the Child Behavior Checklist Attention Problems score and DSM-oriented ADHD subscale. This study is the first to propose the urine microbiome as a potential biomarker for pediatric ADHD.

## 1. Introduction

Attention-deficit hyperactivity disorder (ADHD) is a common neurodevelopmental disorder characterized by inattention, hyperactivity, and impulsivity observed in multiple environments [1]. ADHD is associated with impairments in academic and social functioning and is often accompanied by a range of comorbid psychiatric disorders if left untreated [2,3,4]. The current standard of ADHD screening relies on caregiver and teacher questionnaires, for which possible subjectiveness or bias cannot be ruled out. Conversely, biomarkers can be more objective in diagnosis and at the same time serve as potential therapeutic targets [5]. While several potential biomarkers have been suggested, none have reached a high enough level of evidence for standard clinical usage [6].

Recently, a large number of studies have been exploring the effect of the gut microbiome on psychiatric pathology. As such, the potential use of the human microbiome as a biomarker of various disorders, including mental disorders, is being actively researched. The microbiota–gut–brain axis is the foundation for bidirectional communication between the gut microbiota and the central nervous system. The gut microbiome is associated with various processes such as neuroinflammation, stress axis activation, neurotransmission, blood–brain barrier formation, myelination, and microglial maturation [7,8]. Major mechanisms of bottom-up communication between the brain, gut, and gut microbiome include the neural (operational through direct activation of the enteric nervous system and vagal afferent nerves), endocrinal (through involvement in the development and regulation of the hypothalamus–pituitary–adrenal axis), metabolic (by synthesis of neuroactive molecules), and immune (CNS infiltrating immune cells and systemic inflammation) pathways [9]. Various alterations in gut microbiota have been found in ADHD patients [10,11,12,13,14,15,16], along with associations between dietary or probiotic intervention, microbiota composition [17,18,19], and ADHD-related outcomes, all indicative of possible uses of the human microbiome as a diagnostic tool or biomarker in this patient population.

However, recent studies utilizing next generation sequencing have revealed that the human body contains microbiota not limited to the gut, but also within the genitourinary tract. The urine microbiome, namely “urobiome,” is emerging as an increasingly meaningful branch of the human microbiome, consisting of microorganisms from the lower urinary tract and genital tract [20]. Most research on the urobiome does not touch on child psychiatry and is focused on either the adult population or urological diseases [20,21,22,23,24]. It has previously been found that, in the case of women, 62.5% of intestinal-derived species and 32% of vaginal-derived species are shared with species in urine [25]. This poses the possibility of the unclear origin of the urine microbiome being the gut. Following this idea, Leue et al. reported interrelationship between functional urologic diseases (e.g., overactive bladder, interstitial cystitis/bladder pain syndrome, chronic prostatitis/chronic pelvic pain syndrome) and gastrointestinal disorders and hypothesized the existence of a “bladder–gut–brain axis” to explain this phenomenon [26]. Accordingly, the forementioned evidence of altered gut microbiota in those with ADHD opens the possibility of altered urinary microbiomes as well. In addition, many children with ADHD also experience urinary problems; for instance, one study found that 28% of individuals with ADHD exhibited lower urinary tract symptoms [27]. In multiple studies, the degree of lower urinary tract symptoms, including urinary frequency, pressure, urgency, and overactive bladder syndrome, were significantly higher in patients with ADHD than in healthy individuals. Further, the symptoms worsened along with increased severity of the ADHD. The incidence and severity of lower urinary tract symptoms were also significantly higher in younger children [28].

Regarding psychiatric pathologies, recent studies have indicated that gut microbiota affects neurotransmitters (e.g., dopamine, norepinephrine, and epinephrine), organic acids, and metabolite distributions in the urine. Altered gut microbiota affect the integrity of the gut barrier, leading to variations in the bacterial metabolite absorption and excretion of neurotransmitter precursors into urine [29]. This may be attributed to varied levels of bacterial metabolism within the diagnostic group [10,30,31,32]. In one previous study regarding autistic children, many of the metabolite level alterations observed in the blood and urine were found to be of bacterial origin, including short chain fatty acids (SCFAs), indoles, and lipopolysaccharides (LPS) [30]. Furthermore, in a study by Xiong et al., combination therapy using vancomycin and Bifidobacterium led to lessened autistic symptoms, such as improvement in eye contact and communicative behavior. This therapy resulted in normalized levels of 3-(3-hydroxyphenyl)-3-hydroxypropionic acid, 3-hydroxyphenylacetic acid, and 3-hydroxyhippuric acid in the urine, which provides evidence that supports a possible link between the production of these metabolites within the gut, their presence in the urine, and the pathology of ASD [33]. Another recent study also suggested significantly reduced diversity in the urinary microbiome in patients with higher levels of depression and anxiety [34].

Together, these findings open the discussion of a possible bladder–gut–brain axis taking part in the pathophysiology of ADHD, integrating the interaction between the gastrointestinal and urinary tract microbiome to examine the effects the microbiome or its by-products have on neural processing. Further investigation must be undertaken on whether this axis could involve the same mechanisms of bidirectional communication via neurotransmitter release, immune system stimulation, etc., as the microbiota–gut–brain axis [21].

As opposed to stool samples, urine samples are more convenient to study because the collection is relatively simple, causes less aversion for the subjects, and can be efficiently obtained in large amounts. Moreover, since gram-negative bacteria secrete extracellular membrane vesicles (EVs) to communicate with human host cells, genetic material derived from bacteria-derived EVs indicate specific microbiota that are metabolically or pathologically active [35,36,37]. Therefore, the microbiota reflected from EVs in urine likely represent a more significant proportion of the bodily microbiome, as opposed to profiles obtained from stool samples. Using this method, Lee et al. reported changes in the urine microbiota of individuals with ASD along with a distinct microbiota profile associated with ASD [38].

The urobiome profiles of patients with ADHD have not been reported to date. To fill this gap, we investigated whether bacteria-derived EVs in urine are also useful to rapidly identify microbiota profiles in patients with ADHD. In this study, we investigated and identified the urobiome profiles of Korean boys with ADHD by comparing them with those of healthy male controls. Specifically, the urine microbiota of each participant was analyzed by extracting 16S ribosomal DNA (rDNA) from purified EVs found in urine samples by using 16S next-generation sequencing (NGS).

## 2. Materials and Methods

### 2.1. Subjects and Sample Collection

A total of 33 boys with ADHD were enrolled in the study from January to April 2022 at a child and adolescent psychiatry clinic in a tertiary university hospital in Seoul, Korea. Inclusion criteria were as follows: (1) an age range from 6 to 16 years; (2) a diagnosis of ADHD according to the DSM-V criteria using Mini-International Neuropsychiatric Interview-Kid (MINI-Kid) [39], which is a structured interview tool for children; (3) an IQ of 70 or higher, assessed using the Korean-Wechsler Intelligence Scale for Children-Fourth Edition (K-WISC-IV) [40]; and (4) informed consent provided by both the child and the caregiver. Exclusion criteria included the presence of significant medical or neurological diagnoses, a diagnosis of ASD or intellectual disability, or other comorbid psychiatric disorders; the presence of an alcohol or substance use disorder; the ingestion of pro- or antibiotics within 2 months prior to study participation; the presence of severe case(s) of pediatric immune disease (e.g., atopic dermatitis, asthma, etc.) or gastrointestinal disease in continuous medical treatment; and any specific dietary restrictions (e.g., vegan and vegetarian). The baseline attention problem subscale of the Child Behavior Checklist and ADHD Rating Scale were used to evaluate the severity of the ADHD symptoms. Sex-matched controls were enrolled from Seoul National University Hospital with similar exclusion criteria and informed consent, based on previous literature that the male urobiome does not change significantly with age [20,24,41,42]. First morning midstream urine samples were collected from participants for metagenomic analysis. If unavailable, second morning midstream samples were accepted for collection. Midstream urine (40 mL) was collected into a clean 50 mL urine container, transferred to a conical tube, and stored at −20 °C.

### 2.2. Bacterial and EV Isolation and DNA Extraction from Clinical Samples

Each urine sample was centrifuged at 10,000× *g* for 10 min at 4 °C, and the supernatant was passed through a 0.22 μm membrane filter to eliminate any foreign particles and quantified based on protein concentration. Isolated EVs from each sample (1 μg of protein) were boiled at 100 °C for 40 min and centrifuged at 13,000× *g* for 30 min. Bacterial DNA was extracted from the collected supernatants using a DNA extraction kit (PowerSoil DNA Isolation Kit; MO BIO, Carlsbad, CA, USA) following the manufacturer’s instructions. Isolated DNA was quantified using the QIAxpert system (QIAGEN, Hilden, Germany).

### 2.3. PCR Amplification, Library Construction, and Sequencing of 16S rRNA Gene Variable Regions

Metagenomic analysis of the 16S rRNA gene amplicon was conducted. It was preferred over whole genome sequencing because the 16S rRNA gene is universal in bacteria and can be measured for comparison among all bacteria, while whole genomes of bacteria are of varying sizes and gene duplication, transfer, deletion, fusion, and splitting are common [43,44]. Thirty-five cycles of PCR amplification of the V3-V4 hypervariable regions of the 16S rRNA genes among the prepared bacterial DNA were performed using the primer set 16S_V3_F (5′-TCGTCGGCAGCGTCAGATGTGTATAAGAGACAGCCTACGGGNGGCWGCAG-3′) and 16S_V4_R (5′-GTCTCGTGGGCTCGGAGATGTGTATAAGAGACAGGACTACHVGGGTATCTAATCC-3′) according to MiSeq System guidelines for 16S metagenomic sequencing library preparation (Illumina Inc., San Diego, CA, USA). PCR products were used to construct 16S ribosomal RNA (rRNA) gene libraries for each sample, also following the MiSeq System guidelines. These were then quantified using QIAxpert (QIAGEN, Germany), pooled at an equimolar ratio, and used for pyrosequencing with the MiSeq System (Illumina Inc., San Diego, CA, USA) according to the manufacturer’s protocols. ZymoBIOMICS Microbial Community Standard (Cat # D6300) was used as a positive control to ensure reliable detection.

### 2.4. Analysis of Bacterial Composition in the Microbiome

Paired-end 16S rRNA gene sequences were analyzed via Quantitative Insights into Microbial Ecology (QIIME2 v2021. 4) [34]. Adapter sequences were removed using the Cutadapt software [45]. Reads were filtered for quality and chimeric reads using DADA2 with manual parameters (trim-left-f 0, trim-left-r 0, trunc-len-f 260, trunc-len-r 200, trunc-q 2, max-ee-f 3, and max-ee-r 3) [46]. Taxonomic classification was assigned using a naïve Bayes classifier trained on the extracted V3-V4 region from the SILVA 138 database. SILVA databases have data of 510,984 total sequences including Bacteria, Archaea, and Eukaryota, making it possible to analyze microbial diversity in various environments with extensive comprehensiveness. In addition, because SILVA continuously improves and updates their quality standards, more reliable analysis results are obtainable [47]. All sequences classified as chloroplasts or mitochondria were removed.

### 2.5. Statistical Analysis

Demographic characteristics, including age, baseline Child Behavior Checklist (CBCL) subscale scores, and ADHD-RS scores, were analyzed using SPSS 26.0.0.2. Alpha diversity metrics were assessed using the phyloseq [48] package of R (version 4.1.0). Each sample was rarified with the minimum read value to avoid alpha diversity bias. These metrics were compared based on operational taxonomic units (OTUs), the Chao1 index, Shannon index, and Simpson index. Values were transformed by taking the natural logarithmic value to reduce skewness, and ANCOVAs were performed with age as a control factor. Beta diversity metrics were extracted using the stats package implemented in R. Dimension reduction was conducted using principal coordinate analysis (PCoA) and multiple dimension scale (MDS) to assess the beta diversity between clinical samples based on the Bray–Curtis dissimilarity. Permutational multivariate analysis of variance (PERMANOVA) was used to validate whether either the centroid or the spread of each sample was different between the groups. The bacterial composition of each group was compared at the phylum and genus levels using ANCOVA tests, controlling for age, based on normalized OTU reads of the taxa. Spearman’s correlation between normalized OUT reads of ADHD-specific taxa, and baseline CBCL subscale and ADHD-RS scores was performed using SPSS 26.0.0.2. All statistical analyses were interpreted at a two-sided significance level of 0.05. The study was conducted in accordance with the Declaration of Helsinki, and the protocol was approved by the Institutional Review Board of the Gangnam Severance Hospital, Yonsei University College of Medicine (3-2020-0209).

## 3. Results

### 3.1. Demographic Characteristics

We analyzed urine samples from 72 individuals, including 33 boys with ADHD and 39 healthy adults. Patients with ADHD had a mean age of 9.5 ± 2.5 (range, 6 to 15), and the healthy individuals had a mean age of 21.4 ± 1.3 (range, 19 to 23). The baseline CBCL attention problem subscale, DSM-oriented ADHD t-scores, and ADHD-RS scores of patients with ADHD are displayed in Table 1. According to the best estimate clinician diagnosis, 84.8% (*n* = 28) of the participants with ADHD were of the combined presentation, with the remaining 15.2% being of the predominantly inattentive presentation. Hence, we decided that the ADHD group was homogeneous enough in ADHD subtypes to continue analyses as one whole group.

### 3.2. Comparison of Alpha and Beta Diversity between ADHD and Controls

**Figure 1:**
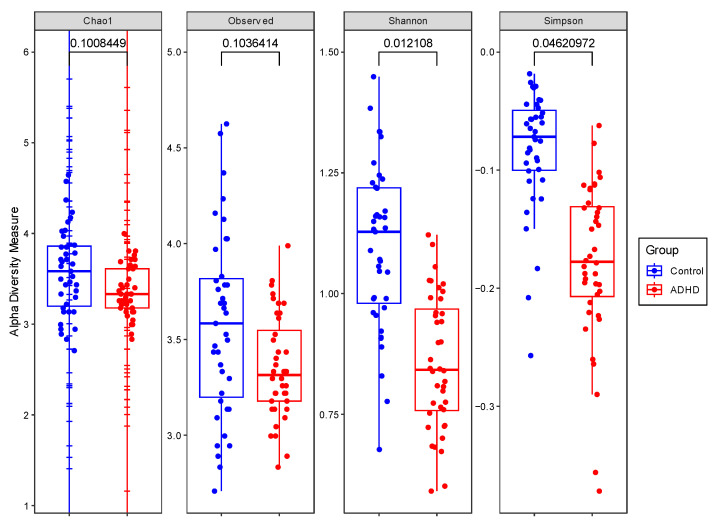
Alpha diversity indices were compared between the ADHD and control group with ANCOVA, controlling for age. From the left, alpha diversity is compared using Chao1, OTUs, Shannon, and Simpson indices. The significant difference in Shannon and Simpson indices implies a greater number and abundance of microbiota in the control group. OTUs and Chao1 did not show significant differences between groups. *p*-values are displayed on the top of each plot.

OTUs, Chao1, Shannon, and Simpson indices were calculated to analyze the alpha diversity of each group. Although the Shannon and Simpson indices showed a significantly greater number of species and their abundance in the healthy individuals (Shannon index, *p* = 0.012; Simpson index, *p* = 0.046), the Chao1 index and OTUs did not (Figure 1).

**Figure 2:**
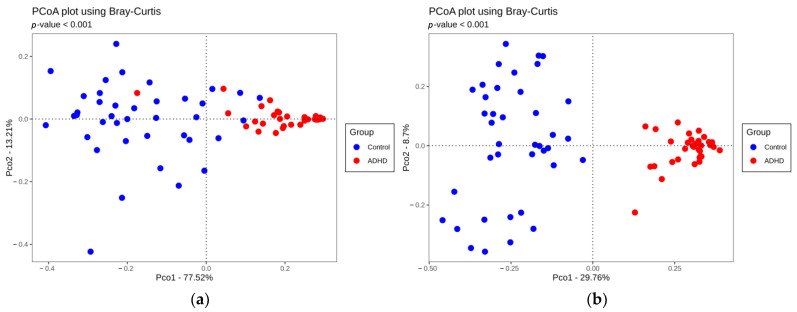
Two-dimensional PCoA plots based on Bray–Curtis similarities. PERMANOVA was performed to analyze the difference in distribution on the PCoA plots. (**a**) Beta diversity at the phylum level, and (**b**) At the genus level.

Bray–Curtis similarity is a popular ecological metric used to examine the similarity between two or more samples based on their bacterial composition, indicated by a value between 0 and 1. The higher the value, the less species they share with each other. The two-dimensional PCoA plot uses Bray–Curtis similarity between each pair of ADHD and control samples and compresses the information to two principal components to visualize beta diversity. The further separated the dots representing each group are, the more different they are in terms of bacterial community conformation. The 2-dimensional PCoA plot showed that the two groups had significantly different bacterial communities at the phylum level and genus level. This pattern was further confirmed by the PERMANOVA analysis performed (*p* < 0.001, Figure 2).

### 3.3. Relative Microbiota Abundance Differences between ADHD and Controls

**Figure 3:**
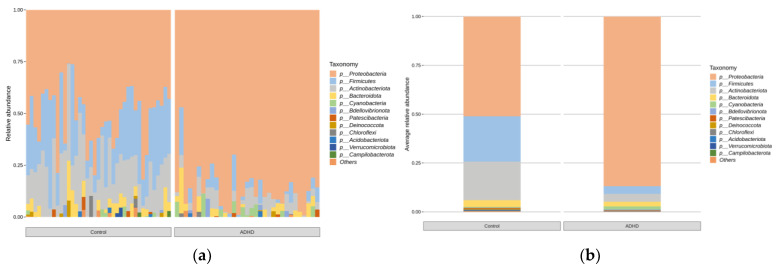
Bar plots of relative abundance of microbes at the phylum level. (**a**) Relative abundance of all samples. (**b**) Average relative abundance of microbes in those with ADHD and healthy individuals.

**Figure 4:**
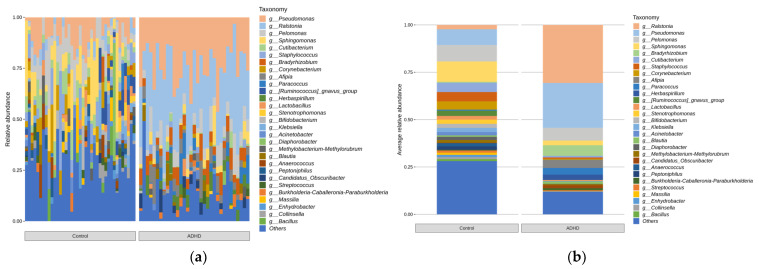
Bar plots of relative abundance of microbes at the genus level. (**a**) Relative abundance of all samples and (**b**) Average relative abundance of microbes in patients with ADHD and healthy individual groups.

Microbial taxonomic analysis was performed to compare the relative abundance of microbiota between the two groups. Figure 3 displays the bar plots for the relative abundance of taxa for each group and bar plots based on the average relative abundance at the phylum level of each group. Equivalent analyses were performed at the genus level (Figure 4).

**Figure 5:**
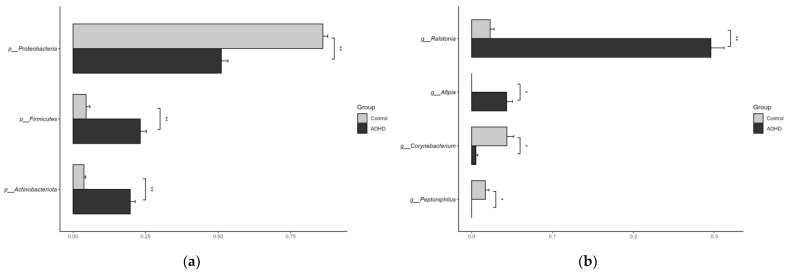
Taxa with significant differences in relative abundance. (**a**) At the phylum level and (**b**) At the genus level. Controlled for age. * *p* < 0.05, ** *p* < 0.01.

Taxa with significant differences in relative abundance at the phylum and genus levels were analyzed using ANCOVAs and controlled for age (*p* < 0.05, Figure 5). The analysis was performed only for taxa with more than 1% of the total reads. Specifically, at the phylum level, *Firmicutes* and *Actinobacteriota* were more abundant in the ADHD group, whereas *Proteobacteria* were more abundant in the control group. At the genus level, *Ralstonia* and *Afipia* were more abundant in the ADHD group, whereas *Corynebacterium* and *Peptoniphilus* were more abundant in the control group.

### 3.4. Correlation between Baseline ADHD Symptom Scores and ADHD-Specific Taxa

We performed Spearman’s correlation analysis to examine whether the number, specifically OTU reads, of certain ADHD-specific taxa was related to ADHD symptom severity (Table 2). Neither *Firmicutes* or *Actinobacteriota* correlated with the CBCL attention problem subscale t-scores. However, at the genus level, *Afipia* showed a correlation with both the attention problem subscale t-score and DSM-oriented ADHD t-score. None of the ADHD-RS scores correlated with the abundance of taxa at the phylum or genus level.

## 4. Discussion

The present study examined the microbiome profile of urine obtained from boys with ADHD by extracting bacterial DNA from bacteria-derived EVs in urine samples. We found significant differences in the urine microbial abundance and diversity between patients with ADHD and those of healthy individuals. The urobiota composition was significantly different in the ADHD group compared with that of the control group. We found a relative abundance of phylum *Firmicutes* and *Actinobacteriota*, along with the genera *Ralstonia* and *Afipia* in boys with ADHD, suggesting the potential use of the urobiome as a biomarker of ADHD. 

### 4.1. Comparison of Alpha and Beta Diversity between ADHD and Controls

We found decreased alpha diversity in the urine microbiome measured using the Shannon and Simpson indices from individuals with ADHD, which is consistent with the results from prior studies on the gut microbiome in ADHD [11,15,17]. However, there were no differences in the OTU reads or Chao1 index, indicating no statistically significant difference in the number of species between the two groups. Moreover, we found different patterns of beta diversity, suggesting that the urine microbial compositions of the two groups were substantially different. Therefore, decreased alpha diversity can be said to have resulted from the differences in urine microbiota distribution between the two groups. 

Regarding the natural increase in the abundance and diversity of the gut microbiome with age [49], we cannot rule out that the age differences between the ADHD group (mean age 9.5 ± 2.5 years) and the healthy group (21.4 ± 1.3 years) may have possibly affected the composition of the urine microbiomes. To address this possibility, we statistically controlled for age in our analyses. While there is evidence to suggest that the female urobiome changes significantly before and after puberty, there is a lack of literature that supports that this is also true for males [20,24,41,42]. Kassiri et al. reported a similar composition and bacterial load in the urobiome of prepubertal males compared with those previously reported in adults [42]. Thus, we considered it reasonable to compare these two groups to identify the urobiome profiles of individuals with ADHD. 

### 4.2. Relative Microbiota Abundance Differences between ADHD and Controls

We found that at the phylum level, there were higher levels of the phyla *Firmicutes* and *Actinobacteriota* along with a relative deficiency of *Proteobacteria* in patients with ADHD compared with healthy individuals. At the genus level, higher levels of genera *Ralstonia* and *Afipia* and a relative deficiency of *Corynebacterium* and *Peptoniphilus* were characteristic of the ADHD group. 

While we believe that EVs extracted from urine represent microbiota from a more variable source of systems [38], it is important to note that the majority of the microbiota in the body are represented by gut microbiota. Studies comparing stool and urine microbiota in individuals without ADHD show that 64% of the species identified from the urine overlap with those identified from stool samples [25]. However, at this stage, there are inconsistencies in reports comparing gut microbiota composition to urine due to the highly variable reports of gut microbiota characteristics in ADHD. Such variability may be due to different sample sizes, inconsistent participant selection criteria, neuropsychological assessments, unidentified confounding factors (e.g., dietary characteristics), the duration of the intervention period, and diverse microbiome analysis techniques (e.g., PCR versus 16S marker gene analysis) [50]. For example, one study found that the abundance of *Bacteroides ovatus* and *Sutterella stercoricanis* in the gut microbiota of individuals were positively correlated with ADHD symptoms [17]. However, another study reported elevated levels of the families *Bacteroidaceae* and *Neisseriaceae* in the gut as possible biomarkers for ADHD and found that the OTU levels of the species *Bacteroides* correlated with parental ratings of hyperactivity and impulsivity [11]. Some studies found significant associations between the order *Clostridiales* and ADHD, although they differed in which specific genus was abundant [12,13,14,16]. In a previous study by Aarts et al., exploring the gut microbiota of individuals with ADHD, *Actinobacteriota* was more abundant in individuals with ADHD, while *Firmicutes* was more abundant in controls. Aarts et al. also stated that *Bifidobacterium*, a genus of the phylum *Actinobacteriota,* seemed responsible for the increase in cyclohexadienyl dehydratase (CDT), an enzyme in the phenylalanine (a dopamine precursor) pathway, in the microbiome of individuals with ADHD. The abundance of CDT was negatively correlated with reward-anticipation responses across the whole brain [10]. 

Our results partly agree with the study by Aarts et al., at the phylum level, as *Actinobateriota* were found to be significantly more abundant in individuals with ADHD. However, on average, the specific genus *Bifidobacterium* comprised only 0.05% of the microbiota of the ADHD group and was found to be significantly lower than that of the controls (*p* = 0.002). Our results are also notably different from those of other studies that have examined the gut microbiome of individuals with ADHD. Since this is the first study to identify and report urobiome characteristics in individuals with ADHD, these discrepancies should be analyzed carefully. This may imply that the microbiome of the urine and gut are substantially different and cannot be compared with each other in the first place, or that a microbiome from a source other than the gut plays a key role in the characteristic urobiome of patients with ADHD. Despite the largely varying results among studies on the microbiome in patients with ADHD, microbiome studies on other neurodevelopmental disorders, such as ASD, show promising potential in this field, with results from multiple studies converging to point towards certain microbiota as biomarkers or possible treatment targets [38,51,52,53,54,55,56]. Therefore, further studies are necessary to examine the relationship between the microbiome of other sources (including the gut) and urine for an accurate insight into this discrepancy and to distinguish a reliable biomarker for ADHD.

### 4.3. Correlation between Baseline Evaluation Scores and ADHD-Specific Taxa

Our correlation results showed that the abundance of the genus *Afipia* had significant correlations with the CBCL attention problems subscale and DSM-oriented ADHD subscale t-scores, but not with the ADHD-RS scores. Although the ADHD-RS is the current gold standard questionnaire used to make ADHD diagnoses, the correlation seen in the CBCL attention problems subscale is still notable. To the best of our knowledge, no previous studies have determined the effect of the genus *Afipia* and its function on the central nervous system. Therefore, we could not exclude the possibility that this genus could play a pathogenic role in individuals with ADHD. Given that the genus is mainly associated with infections [57], there is speculative potential for it to affect the immune system, which may have implications for ADHD-related symptomatology. Further studies using diverse means to evaluate ADHD symptoms may be useful in identifying additional microbiota specifically related to symptom severity. 

### 4.4. Limitations

Our study had several limitations. First, we could not obtain an age-matched sample and thus had to conduct the analyses under the assumption that the urobiome of males would not change substantially with age, while also statistically controlling for age as a covariate in our analyses. Our results should be interpreted with care in terms of clinical implications as it is a limited primary investigation on this topic. Future studies with direct comparisons between ADHD individuals and their age-matched controls within each sex are required for a more accurate analysis. Second, we did not control for certain lifestyles, such as engagement in sexual interests, that may contribute to the microbiome obtained from midstream urine. While the risk of contamination in midstream urine samples in men is lower than in women due to anatomic differences [25], the possibility is still present. Further studies incorporating confidential questions at baseline to take this factor into consideration could be beneficial. Third, we did not consider the possible diurnal variation of urinary microbiomes or microbial by-products. Instead, we controlled for this factor by collecting first or second morning midstream urine only. Fourth, we did not conduct analyses of the possible functions of each phylum or genus. Therefore, we could not define how different microbiome compositions may have contributed to differing pathways of individuals with ADHD and their pathogenesis. Additional research that uses more advanced analyses, such as using Phylogenetic Investigation of Communities by Reconstruction of Unobserved States (PICRUSt), would provide further insight into the bladder–brain axis, and its role in patients with ADHD. Fifth, our study participants were entirely male, thus our study results should be interpreted with caution when generalizing to other groups, such as females with ADHD. In this study, we aimed to provide the best sex-matched analysis possible, as microbial and/or metabolic profiles in the urine of individuals with ADHD can significantly differ by sex [58,59]. Further study with the rigorous recruitment of female participants, despite the low prevalence of ADHD in females [60], is warranted. Finally, our study compared only a small number of individuals with ADHD and controls. A study with a larger sample size would be beneficial for the better identification of urobiome profiles.

## 5. Conclusions

To the best of our knowledge, our study is the first to show that the urobiome of Korean children with ADHD is significantly different from that of healthy individuals. We demonstrate that bacteria-derived EVs in the urine can be a reliable source of urine micro with ADHD from healthy children. Although this portrays the potential use of urine samples as a biomarker for pediatric ADHD, further research is needed in order to implement our findings directly to clinical usage.

## Figures and Tables

**Table 1:** Baseline CBCL and ADHD-RS results of ADHD individuals.

Scales	Mean ± SD
CBCL	
Attention problems subscale ^a^	61.30 ± 7.16
DSM-oriented ADHD subscale ^a^	64.30 ± 11.91
ADHD-RS	
Inattention	11.03 ± 5.35
Hyperactivity/impulsivity	10.55 ± 5.18
Total	21.58 ± 9.92

^a^ CBCL subscale t-scores.

**Table 2:** Correlation between baseline evaluation and normalized OTU reads.

Phylum Level	*Firmicutes* ^b^	*Actinobacteriota* ^b^
CBCL		
Attention	0.154 (0.392)	0.138 (0.445)
Problems		
Subscale ^a^		
DSM oriented	0.167 (0.352)	0.157 (0.383)
ADHD		
Subscale ^a^		
ADHD-RS		
Inattention	−0.057 (0.154)	0.003 (0.987)
Hyperactivity	0.025 (0.889)	0.114 (0.528)
Total	−0.016 (0.930)	0.043 (0.813)
**Genus level**	***Ralstonia*** ^b^	***Afipia*** ^b^
CBCL		
Attention	−0.289 (0.103)	0.427 × (0.013)
Problems		
Subscale ^a^		
DSM	−0.339 (0.054)	0.375 × (0.032)
oriented		
ADHD		
subscale ^a^		
ADHD-RS		
Inattention	−0.012 (0.947)	−0.076 (0.676)
H/I	0.049 (0.786)	−0.149 (0.409)
Total	0.015 (0.936)	−0.093 (0.607)

^a^ CBCL subscale *t*-score. ^b^ Displayed as *r* (*p*-value).

## Data Availability

For data on the ADHD group, the data presented are openly available in the Sequence Read Archive (SRA) repository, National Center for Biotechnology Information (NCBI) at https://www.ncbi.nlm.nih.gov/bioproject/PRJNA938268, accessed on 26 July 2023, BioProject accession number PRJNA938268. For data on the healthy male adult group, restrictions apply to the availability. Data were obtained from Eun Ju Cho, Seoul National University and are available from the authors with the permission of Eun Ju Cho.

## References

[1] American Psychiatric Association (2013). Diagnostic and Statistical Manual of Mental Disorders.

[2] Biederman J., Newcorn J., Sprich S. (1991). Comorbidity of Attention Deficit Hyperactivity Disorder with Conduct, Depressive, Anxiety, and Other Disorders. Am. J. Psychiatry.

[3] Spencer T.J., Biederman J., Mick E. (2007). Attention-Deficit/Hyperactivity Disorder: Diagnosis, Lifespan, Comorbidities, and Neurobiology. Ambul. Pediatr..

[4] Biederman J., Monuteaux M.C., Doyle A.E., Seidman L.J., Wilens T.E., Ferrero F., Morgan C.L., Faraone S.V. (2004). Impact of Executive Function Deficits and Attention-Deficit/Hyperactivity Disorder (ADHD) on Academic Outcomes in Children. J. Consult. Clin. Psychol..

[5] Chen H., Yang Y., Odisho D., Wu S., Yi C., Oliver B.G. (2023). Can Biomarkers Be Used to Diagnose Attention Deficit Hyperactivity Disorder?. Front. Psychiatry.

[6] Hebebrand J., Antel J. (2014). Squaring the Circle? On the Search for Circulating Biomarkers in Polygenic Psychiatric Disorders. Eur. Child Adolesc. Psychiatry.

[7] Sherwin E., Dinan T.G., Cryan J.F. (2018). Recent Developments in Understanding the Role of the Gut Microbiota in Brain Health and Disease. Ann. N. Y. Acad. Sci..

[8] Fung T.C., Olson C.A., Hsiao E.Y. (2017). Interactions between the Microbiota, Immune and Nervous Systems in Health and Disease. Nat. Neurosci..

[9] Wang Y., Kasper L.H. (2014). The Role of Microbiome in Central Nervous System Disorders. Brain Behav. Immun..

[10] Aarts E., Ederveen T.H.A., Naaijen J., Zwiers M.P., Boekhorst J., Timmerman H.M., Smeekens S.P., Netea M.G., Buitelaar J.K., Franke B. (2017). Gut Microbiome in ADHD and Its Relation to Neural Reward Anticipation. PLoS ONE.

[11] Prehn-Kristensen A., Zimmermann A., Tittmann L., Lieb W., Schreiber S., Baving L., Fischer A. (2018). Reduced Microbiome Alpha Diversity in Young Patients with ADHD. PLoS ONE.

[12] Jiang H.-Y., Zhou Y.-Y., Zhou G.-L., Li Y.-C., Yuan J., Li X.-H., Ruan B. (2018). Gut Microbiota Profiles in Treatment-Naïve Children with Attention Deficit Hyperactivity Disorder. Behav. Brain Res..

[13] Szopinska-Tokov J., Dam S., Naaijen J., Konstanti P., Rommelse N., Belzer C., Buitelaar J., Franke B., Bloemendaal M., Aarts E. (2020). Investigating the Gut Microbiota Composition of Individuals with Attention-Deficit/Hyperactivity Disorder and Association with Symptoms. Microorganisms.

[14] Wan L., Ge W.-R., Zhang S., Sun Y.-L., Wang B., Yang G. (2020). Case-Control Study of the Effects of Gut Microbiota Composition on Neurotransmitter Metabolic Pathways in Children With Attention Deficit Hyperactivity Disorder. Front. Neurosci..

[15] Casas L., Karvonen A.M., Kirjavainen P.V., Täubel M., Hyytiäinen H., Jayaprakash B., Lehmann I., Standl M., Pekkanen J., Heinrich J. (2019). Early Life Home Microbiome and Hyperactivity/Inattention in School-Age Children. Sci. Rep..

[16] Cheng S., Han B., Ding M., Wen Y., Ma M., Zhang L., Qi X., Cheng B., Li P., Kafle O.P. (2020). Identifying Psychiatric Disorder-Associated Gut Microbiota Using Microbiota-Related Gene Set Enrichment Analysis. Brief. Bioinform..

[17] Wang L.J., Yang C.Y., Chou W.J., Lee M.J., Chou M.C., Kuo H.C., Yeh Y.M., Lee S.Y., Huang L.H., Li S.C. (2020). Gut Microbiota and Dietary Patterns in Children with Attention-Deficit/Hyperactivity Disorder. Eur. Child Adolesc. Psychiatry.

[18] Pärtty A., Kalliomäki M., Wacklin P., Salminen S., Isolauri E. (2015). A Possible Link between Early Probiotic Intervention and the Risk of Neuropsychiatric Disorders Later in Childhood: A Randomized Trial. Pediatr. Res..

[19] Stevens A.J., Purcell R.V., Darling K.A., Eggleston M.J.F., Kennedy M.A., Rucklidge J.J. (2019). Human Gut Microbiome Changes during a 10 Week Randomised Control Trial for Micronutrient Supplementation in Children with Attention Deficit Hyperactivity Disorder. Sci. Rep..

[20] Wojciuk B., Salabura A., Grygorcewicz B., Kędzierska K., Ciechanowski K., Dołęgowska B. (2019). Urobiome: In Sickness and in Health. Microorganisms.

[21] Peyronnet B., Mironska E., Chapple C., Cardozo L., Oelke M., Dmochowski R., Amarenco G., Gamé X., Kirby R., van der Aa F. (2019). A Comprehensive Review of Overactive Bladder Pathophysiology: On the Way to Tailored Treatment. Eur. Urol..

[22] Tang J. (2017). Microbiome in the Urinary System—A Review. AIMS Microbiol..

[23] Gerber D., Forster C.S., Hsieh M. (2018). The Role of the Genitourinary Microbiome in Pediatric Urology: A Review. Curr. Urol. Rep..

[24] Kawalec A., Zwolińska D. (2022). Emerging Role of Microbiome in the Prevention of Urinary Tract Infections in Children. Int. J. Mol. Sci..

[25] Perez-Carrasco V., Soriano-Lerma A., Soriano M., Gutiérrez-Fernández J., Garcia-Salcedo J.A. (2021). Urinary Microbiome: Yin and Yang of the Urinary Tract. Front. Cell. Infect. Microbiol..

[26] Leue C., Kruimel J., Vrijens D., Masclee A., van Os J., van Koeveringe G. (2017). Functional Urological Disorders: A Sensitized Defence Response in the Bladder-Gut-Brain Axis. Nat. Rev. Urol..

[27] de Almeida Vasconcelos M.M., Netto J.M.B., Arana I.E., Teixeira I.B., Lima E.M., Carvalho T.A., de Bessa Junior J., de Carvalho Mrad F.C. (2021). Association between Attention Deficit Hyperactivity Disorder and Lower Urinary Tract Symptoms in Children and Adolescents in a Community Setting. Int. Braz. J. Urol..

[28] Mahjani B., Koskela L.R., Mahjani C.G., Janecka M., Batuure A., Hultman C.M., Reichenberg A., Buxbaum J.D., Akre O., Grice D.E. (2022). Systematic Review and Meta-Analysis: Relationships between Attention-Deficit/Hyperactivity Disorder and Urinary Symptoms in Children. Eur. Child Adolesc. Psychiatry.

[29] Richarte V., Sánchez-Mora C., Corrales M., Fadeuilhe C., Vilar-Ribó L., Arribas L., Garcia E., Rosales-Ortiz S.K., Arias-Vasquez A., Soler-Artigas M. (2021). Gut Microbiota Signature in Treatment-Naïve Attention-Deficit/Hyperactivity Disorder. Transl. Psychiatry.

[30] Srikantha P., Mohajeri M.H. (2019). The Possible Role of the Microbiota-Gut-Brain-Axis in Autism Spectrum Disorder. Int. J. Mol. Sci..

[31] Daneberga Z., Nakazawa-Miklasevica M., Berga-Svitina E., Murmane D., Isarova D., Cupane L., Masinska M., Nartisa I., Lazdane A., Miklasevics E. (2021). Urinary Organic Acids Spectra in Children with Altered Gut Microbiota Composition and Autistic Spectrum Disorder. Nord. J. Psychiatry.

[32] Gevi F., Belardo A., Zolla L. (2020). A Metabolomics Approach to Investigate Urine Levels of Neurotransmitters and Related Metabolites in Autistic Children. Biochim. Biophys. Acta.

[33] Xiong X., Liu D., Wang Y., Zeng T., Peng Y. (2016). Urinary 3-(3-Hydroxyphenyl)-3-Hydroxypropionic Acid, 3-Hydroxyphenylacetic Acid, and 3-Hydroxyhippuric Acid Are Elevated in Children with Autism Spectrum Disorders. Biomed. Res. Int..

[34] Wu P., Chen Y., Zhao J., Zhang G., Chen J., Wang J., Zhang H. (2017). Urinary Microbiome and Psychological Factors in Women with Overactive Bladder. Front. Cell. Infect. Microbiol..

[35] Kang C.-S., Ban M., Choi E.-J., Moon H.-G., Jeon J.-S., Kim D.-K., Park S.-K., Jeon S.G., Roh T.-Y., Myung S.-J. (2013). Extracellular Vesicles Derived from Gut Microbiota, Especially Akkermansia Muciniphila, Protect the Progression of Dextran Sulfate Sodium-Induced Colitis. PLoS ONE.

[36] Yoo J.Y., Rho M., You Y.A., Kwon E.J., Kim M.H., Kym S., Jee Y.K., Kim Y.K., Kim Y.J. (2016). 16S RRNA Gene-Based Metagenomic Analysis Reveals Differences in Bacteria-Derived Extracellular Vesicles in the Urine of Pregnant and Non-Pregnant Women. Exp. Mol. Med..

[37] Jang S.C., Kim S.R., Yoon Y.J., Park K.S., Kim J.H., Lee J., Kim O.Y., Choi E.J., Kim D.K., Choi D.S. (2015). In Vivo Kinetic Biodistribution of Nano-Sized Outer Membrane Vesicles Derived from Bacteria. Small.

[38] Lee Y., Park J.Y., Lee E.H., Yang J., Jeong B.R., Kim Y.K., Seoh J.Y., Lee S.H., Han P.L., Kim E.J. (2017). Rapid Assessment of Microbiota Changes in Individuals with Autism Spectrum Disorder Using Bacteria-Derived Membrane Vesicles in Urine. Exp. Neurobiol..

[39] Sheehan D.V., Sheehan K.H., Shytle R.D., Janavs J., Bannon Y., Rogers J.E., Milo K.M., Stock S.L., Wilkinson B. (2010). Reliability and Validity of the Mini International Neuropsychiatric Interview for Children and Adolescents (MINI-KID). J. Clin. Psychiatry.

[40] Kwak K.J., Park H.W., Kim C.T. (2002). A Study for the Standardization of Korean WISC-3 (1). Korean J. Dev. Psychol..

[41] Storm D.W., Copp H.L., Halverson T.M., Du J., Juhr D., Wolfe A.J. (2022). A Child’s Urine Is Not Sterile: A Pilot Study Evaluating the Pediatric Urinary Microbiome. J. Pediatr. Urol..

[42] Kassiri B., Shrestha E., Kasprenski M., Antonescu C., Florea L.D., Sfanos K.S., Wang M.H. (2019). A Prospective Study of the Urinary and Gastrointestinal Microbiome in Prepubertal Males. Urology.

[43] Clarridge J.E. (2004). Impact of 16S RRNA Gene Sequence Analysis for Identification of Bacteria on Clinical Microbiology and Infectious Diseases. Clin. Microbiol. Rev..

[44] Bolyen E., Rideout J.R., Dillon M.R., Bokulich N.A., Abnet C.C., Al-Ghalith G.A., Alexander H., Alm E.J., Arumugam M., Asnicar F. (2019). Reproducible, Interactive, Scalable and Extensible Microbiome Data Science Using QIIME 2. Nat. Biotechnol..

[45] Martin M. (2011). Cutadapt Removes Adapter Sequences from High-Throughput Sequencing Reads. EMBnet J..

[46] Callahan B.J., McMurdie P.J., Rosen M.J., Han A.W., Johnson A.J.A., Holmes S.P. (2016). DADA2: High-Resolution Sample Inference from Illumina Amplicon Data. Nat. Methods.

[47] Quast C., Pruesse E., Yilmaz P., Gerken J., Schweer T., Yarza P., Peplies J., Glöckner F.O. (2013). The SILVA Ribosomal RNA Gene Database Project: Improved Data Processing and Web-Based Tools. Nucleic Acids Res..

[48] McMurdie P.J., Holmes S., Jordan G., Chamberlain S. (2023). Phyloseq: Handling and Analysis of High-Throughput Microbiome Census Data. https://rdrr.io/bioc/phyloseq/.

[49] Yatsunenko T., Rey F.E., Manary M.J., Trehan I., Dominguez-Bello M.G., Contreras M., Magris M., Hidalgo G., Baldassano R.N., Anokhin A.P. (2012). Human Gut Microbiome Viewed across Age and Geography. Nature.

[50] Checa-Ros A., Jeréz-Calero A., Molina-Carballo A., Campoy C., Muñoz-Hoyos A. (2021). Current Evidence on the Role of the Gut Microbiome in ADHD Pathophysiology and Therapeutic Implications. Nutrients.

[51] Ha S., Oh D., Lee S., Park J., Ahn J., Choi S., Cheon K.-A. (2021). Altered Gut Microbiota in Korean Children with Autism Spectrum Disorders. Nutrients.

[52] de Angelis M., Piccolo M., Vannini L., Siragusa S., de Giacomo A., Serrazzanetti D.I., Cristofori F., Guerzoni M.E., Gobbetti M., Francavilla R. (2013). Fecal Microbiota and Metabolome of Children with Autism and Pervasive Developmental Disorder Not Otherwise Specified. PLoS ONE.

[53] Adams J.B., Johansen L.J., Powell L.D., Quig D., Rubin R.A. (2011). Gastrointestinal Flora and Gastrointestinal Status in Children with Autism -- Comparisons to Typical Children and Correlation with Autism Severity. BMC Gastroenterol..

[54] Wang L., Christophersen C.T., Sorich M.J., Gerber J.P., Angley M.T., Conlon M.A. (2011). Low Relative Abundances of the Mucolytic Bacterium Akkermansia Muciniphila and Bifidobacterium Spp. in Feces of Children with Autism. Appl. Environ. Microbiol..

[55] Strati F., Cavalieri D., Albanese D., de Felice C., Donati C., Hayek J., Jousson O., Leoncini S., Renzi D., Calabrò A. (2017). New Evidences on the Altered Gut Microbiota in Autism Spectrum Disorders. Microbiome.

[56] Finegold S.M., Dowd S.E., Gontcharova V., Liu C., Henley K.E., Wolcott R.D., Youn E., Summanen P.H., Granpeesheh D., Dixon D. (2010). Pyrosequencing Study of Fecal Microflora of Autistic and Control Children. Anaerobe.

[57] Chomel B.B., Kasten R.W., Stuckey M.J., Breitschwerdt E.B., Maggi R.G., Henn J.B., Koehler J.E., Chang C. (2014). Experimental Infection of Cats with Afipia Felis and Various Bartonella Species or Subspecies. Vet. Microbiol..

[58] Swann J.R., Diaz Heijtz R., Mayneris-Perxachs J., Arora A., Isaksson J., Bölte S., Tammimies K. (2023). Characterizing the Metabolomic Signature of Attention-Deficit Hyperactivity Disorder in Twins. Neuropharmacology.

[59] Jašarević E., Morrison K.E., Bale T.L. (2016). Sex Differences in the Gut Microbiome–Brain Axis across the Lifespan. Philos. Trans. R. Soc. B.

[60] Mowlem F.D., Rosenqvist M.A., Martin J., Lichtenstein P., Asherson P., Larsson H. (2019). Sex Differences in Predicting ADHD Clinical Diagnosis and Pharmacological Treatment. Eur. Child Adolesc. Psychiatry.

